# Influenza Type B Complicates a Previously Undiagnosed Case of Pericarditis

**DOI:** 10.7759/cureus.30810

**Published:** 2022-10-28

**Authors:** Keerti Ivaturi, Valerie Tsukhai, Wail M Hassan

**Affiliations:** 1 Biomedical Sciences, University of Missouri Kansas City School of Medicine, Kansas City, USA

**Keywords:** differential diagnosis of acute chest pain, infectious pericarditis, non-effusive pericarditis, missed case of pericarditis, echocardiogram (echo), influenza b, pericarditis

## Abstract

We report the first case of pericarditis exacerbation due to influenza B viral infection while emphasizing the importance of cardiac magnetic resonance (CMR) for the timely diagnosis and ruling out of non-effusive pericarditis in a patient with compatible, unexplained chest pain. The patient presented with left-sided chest pain that was partially relieved by leaning backward and noted persistent fatigue for several days. Pericardial friction rub, electrocardiogram (ECG), and echocardiogram abnormalities were not detected. After discharge on the morning following admission, fatigue and fever several minutes after physical exertion continued. The patient contracted influenza type B, leading to pneumonia and a second hospitalization, during which echocardiography showed moderate pericardial effusion. We conclude that the patient had pericarditis on the first admission because other compatible causes of chest pain were ruled out, symptoms were compatible with non-effusive pericarditis and could not be ruled out since CMR was not done, and the patient tested positive during his second admission for multiple known etiologic agents of pericarditis. We highlight the importance of CMR in screening patients presenting with chest pain of unknown origin to facilitate early detection and intervention.

## Introduction

The pericardium, a sac-like structure that surrounds the heart, is composed of an outer fibrous monolayer (fibrosa or fibrous parietal pericardium) surrounding a serous double layer (serosa) [1-3]. The serosa, in turn, is composed of the outer parietal layer (serous parietal pericardium) and the innermost layer of the pericardium, the visceral layer (serous visceral pericardium), which is intimately associated with the myocardium and pericardial adipose tissue [2,3]. The serous visceral pericardium along with the pericardial adipose tissue makes up the epicardium [2]. The space enclosed between the two serous layers known as the pericardial space is filled with an ultrafiltrate of plasma known as the pericardial fluid. In a normal adult, there is 10 to 50 mL of pericardial fluid [2,4]. Larger volumes of pericardial fluid may accumulate in pericarditis, a condition known as pericardial effusion [5].

Pericarditis refers to the inflammation of the pericardium [1]. The European Society of Cardiology, American College of Cardiology, American Association for Thoracic Surgery, American Heart Association, American Society of Echocardiography, American Society of Nuclear Cardiology, Heart Rhythm Society, Society for Cardiovascular Angiography and Interventions, Society of Cardiovascular Computed Tomography, Society for Cardiovascular Magnetic Resonance, and the Society of Thoracic Surgeons collectively define the diagnostic criteria of acute pericarditis as any two of the following: (1) typical chest pain, (2) pericardial friction rub, (3) suggestive electrocardiogram (ECG) changes, and (4) a new or worsening pericardial effusion [1,6-8].

Pericarditis may present as an acute, subacute, recurrent, or chronic condition [1]. It could be effusive (i.e., accompanied by pericardial effusion) or non-effusive [9]. Accumulated fluid in effusive pericarditis may exert enough pressure on the myocardium to impair cardiac hemodynamics leading to diminished cardiac output and tamponade [9]. The pericardial sac may expand to accommodate a slowly developing effusion, thereby, alleviating the pressure exerted on the heart muscle and preserving cardiac hemodynamics [9]. However, a more rapid or extensive increase in the pericardial fluid volume may exceed the accommodative capacity of pericardial hypertrophy and is, therefore, more likely to trigger a cardiac tamponade [5,9].

The diagnosis of acute pericarditis relies chiefly on clinical presentation, physical examination, and imaging studies. Precordial or retrosternal chest pain is the cardinal symptom of acute pericarditis, which may radiate to the trapezius ridge, neck, left shoulder, or arms [3,8,10]. Pain quality is most frequently sharp [3,8,10] but could also be dull or throbbing [10]. Unlike in myocardial infarction (MI), chest pain in a typical case of acute pericarditis is positional with lying in the supine position, inspiration, and coughing causing exacerbation while leaning forward reduces the pain [3,8,10,11]. Other symptoms include fever, tachycardia, dyspnea, and cough [3,8,10]. Useful diagnostic biomarkers that may aid the diagnosis include elevated serum C-reactive protein (CRP), erythrocyte sedimentation rate (ESR), leukocyte count [3,6,8,10,11], and in the presence of concurrent myocardial injury, elevated serum troponin [6,8,10].

Typical ECG abnormalities are found in approximately 60% of pericarditis patients [10]. These patients may experience up to four distinct stages of ECG findings [3,10]. Stage I is defined by a widespread, concave ST segment elevation and/or PR segment depression [3,6,10,12]. This stage lasts for hours to days and is pathognomonic of pericarditis [3,10]. Stage II, which develops within the first week of illness [3], is marked by the normalization of the ST and PR segments [3,10]. Stage III is characterized by a T wave inversion, which normalizes in stage IV [10]. Given the temporal evolution of typical ECG findings, delayed presentation to the healthcare facility may interfere with the capture of such findings during an acute episode [3]. Furthermore, approximately 40% of patients present with no or atypical, non-pathognomonic ECG abnormalities [3,10]. Therefore, the absence of typical ECG findings cannot rule out pericarditis.

Current multi-society guidelines recommend transthoracic echocardiography (TTE) for an initial screening for pericarditis [7,8,13], and to consider cardiovascular magnetic resonance (CMR) and cardiac computed tomography (CCT) as ancillary tests if acute pericarditis is suspected but has not been detected using echocardiography [7,8]. Transthoracic echocardiography is, therefore, the most commonly used imaging technique in the diagnosis of pericarditis, and often is the only one used [10]. Advantages of TTE include cost effectiveness, broad availability, portability, and promptness of obtaining the results [13,14]. Echocardiography is useful in the detection and quantification of pericardial effusion and in the evaluation of cardiac hemodynamics [10,13]. Although it does not directly detect inflammation of the myocardium, echocardiography may aid in the detection of myocarditis-associated ventricular dysfunction [10]. Echocardiography does not reliably detect inflammation of the pericardium or myocardium, nor does it detect thickening or calcification of the pericardium [13]. A CCT enables the detection of calcification and thickening of the pericardium but is limited in detecting inflammation [13]. Inflammation of the pericardium or myocardium is best illustrated by CMR [13,14]. Therefore, the diagnosis of acute non-effusive pericarditis in the absence of pathognomonic ECG findings may require CCT or CMR. A 2013 case report discussed a patient who presented with dyspnea and no other complaints, ECG findings, or friction rub [15]. The diagnosis of acute pericarditis was only made after CCT showed thickening of the pericardium and follow-up CMR confirmed diffuse pericardial enhancement consistent with pericarditis [15].

Currently, there are no biomarkers or laboratory tests that can definitively diagnose acute pericarditis [10]. However, pericardial inflammation causes a systemic elevation of inflammatory markers; increased ESR, high CRP, and leukocytosis are almost universal in acute pericarditis [3]. Therefore, clinicians may use inflammatory biomarkers to support clinical, ECG, and diagnostic imaging findings indicative of acute pericarditis and to monitor the response to therapy in individual patients [3,10].

Diverse etiologies may lead to inflammation of the pericardium. These can be broadly classified into infectious and non-infectious etiologies [6]. Non-infectious causes include autoimmunity, MI, and malignancies [6,8]. Numerous microorganisms have been linked to pericarditis including viral, bacterial, fungal, and parasitic agents [3]. The frequency of implication of various causative agents in pericarditis remains poorly defined and varies by geographical location and patient population [6]. Generally, it is believed that idiopathic disease and viral agents cause most cases of acute pericardial disease in industrialized nations, while tuberculosis is the number one cause worldwide and in tuberculosis-endemic areas [3,6,11]. However, in a French study conducted between 2007 and 2012, 54% (29/53) of the confirmed infectious etiologies were caused by bacterial agents [16]. Bacterial agents, other than *Mycobacterium tuberculosis*, that are commonly implicated in pericarditis include *Streptococcus pneumoniae* and *Staphylococcus aureus* [3,16]. Other bacterial causes include *Legionella*, *Borrelia burgdorferi*,* Haemophilus*, *Rickettsia*, *Chlamydophila psittaci*, *Mycoplasma pneumoniae*, *Nocardia*, *Treponema pallidum*, *Coxiella burnetii*, *Neisseria meningitidis*, *Listeria*, and *Actinobacillu*s [3,17,18]. Viral agents include human immunodeficiency syndrome virus, coxsackievirus, echovirus, other enteroviruses, influenza types A and B viruses, parainfluenza viruses, measles virus, respiratory syncytial virus, cytomegalovirus, Epstein-Barr virus, herpes simplex virus, varicella-zoster virus, adenovirus, hepatitis A, B, and C viruses, and parvovirus B19 [3]. Fungal and parasitic agents include *Candida*, *Aspergillus*, *Histoplasma*,* Coccidioides*, *Blastomyces*, *Echinococcus*, *Entamoeba histolytica*, and *Toxoplasma gondii* [3].

It is important to note that each infectious agent of the pericardium may also affect the myocardium as well. Therefore, pericarditis and myocarditis may co-exist and/or may develop from one another. Additionally, data suggest that 20.25% of individuals with negative ischemic evaluation and chest pain had actually presented with pericarditis that was undetected by standard diagnostic criteria for pericarditis [19]. In a study by Mikolich et al., out of 44 patients with CMR-documented pericarditis, only five (11.3%) presented with pericardial effusion, 22 (50%) had a pericardial rub, and 24 (54.5%) had ECG changes suggestive of pericarditis [12]. These data clearly indicate that without performing CMR, the diagnosis of non-effusive pericarditis will likely be missed in many patients. 

## Case presentation

We could only find two published reports of pericarditis caused by influenza virus B [20,21]. We have also found a case report linking pericarditis to influenza vaccination [22], a linkage that has been supported by other literature [23]. To the best of our knowledge, this is the first case report of pericarditis exacerbation due to influenza B infection. The atypical presentation of this case, coupled with the absence of any pathognomonic findings, makes it ideally suited to illustrate the danger of skipping CMR in a patient with a suggestive-whether typical or atypical-chest pain that could not otherwise be explained.

Case one

The patient is a 49-year-old male of Arab/Egyptian origin who developed allergies to his pet guinea pig approximately one year before presenting to the emergency department. He believed his allergies had predisposed him to frequent upper respiratory infections (URIs), including frequent episodes of sinusitis, some of which were febrile, during the past year. During the week prior to the emergency department visit, the patient had a URI composed of a productive cough, nasal congestion, and chest pain compatible with bronchitis. His URI was treated with azithromycin 500 mg every day (QD) for three days, which was completed the day prior to the emergency department visit. The patient presented to the emergency department in early October with severe, left-sided chest pain and difficulty breathing. He described his chest pain as severe crushing and pressing pain that radiated to his neck, jaw, and right shoulder. His chest pain was exacerbated by inspiration and coughing and was alleviated by leaning backward as opposed to forward as seen in typical cases of acute pericardial disease. The patient did not have a history of hypertension except for occasional, brief, stress-associated spikes that occurred throughout the two decades prior to his illness. The patient’s father died at the age of 51 years due to acute MI, and had hyperlipidemia for which he had been taking atorvastatin 10 mg QD for the past several years. Physical examination revealed an alert and oriented patient with moderate chest pain, a temperature of 98.5˚F (36.9˚C), heart rate of 104 beats/minute, respiratory rate of 20 breaths/minute, blood pressure of 134/93 mmHg, mean arterial pressure of 107 mmHg, and oxygen saturation of 98% on room air. Lungs were clear to auscultation bilaterally, heart rhythm was regular, the abdomen was soft and non-tender to palpation, and no edema was noted. Laboratory investigations were significant for leukocytosis (21.2 109/L), neutrophilia (18 109/L, 85%), slight monocytosis (1.3 109/L, 6%), and slightly elevated D-dimer (520 ng/mL fibrinogen equivalent units) (Table 1).

**Table 1:** Laboratory investigation results of the patient on the day of admission MCV: Mean corpuscular volume, MCH: Mean corpuscular hemoglobin, MCHC: Mean corpuscular hemoglobin concentration

Parameter	Results	Reference range
White blood cells (× 10^9^/L)	21.2	4.5-11.0
Red blood cells (× 10^12^/L)	5.66	4.3-5.9
Hemoglobin (g/dL)	16.6	13.5-17.5
Hematocrit (%)	50	41-53
MCV (fl)	88	80-100
MCH (pg)	29	25.4-34.6
MCHC (g/dL)	34	31%-36
Platelets (× 10^9^/L)	299	150-450
Neutrophils (× 10^9^/L)	18.0	2.5-8.0
Lymphocytes (× 10^9^/L)	1.2	1.0-4.0
Monocytes (× 10^9^/L)	1.3	0.1-0.7
Eosinophils (× 10^9^/L)	0.5	0.05-0.50
Basophils (× 10^9^/L)	0.1	0.025-0.100
Neutrophils (%)	85	55-70
Lymphocytes (%)	6	20-40
Monocytes (%)	6	2-8
Eosinophils (%)	2	1-4
Basophils (%)	0	0.5-1
Troponin T (ng/mL)	<0.02	<0.02
D-dimer (ng/mL fibrinogen equivalent units)	520	<500

**Figure 1:**
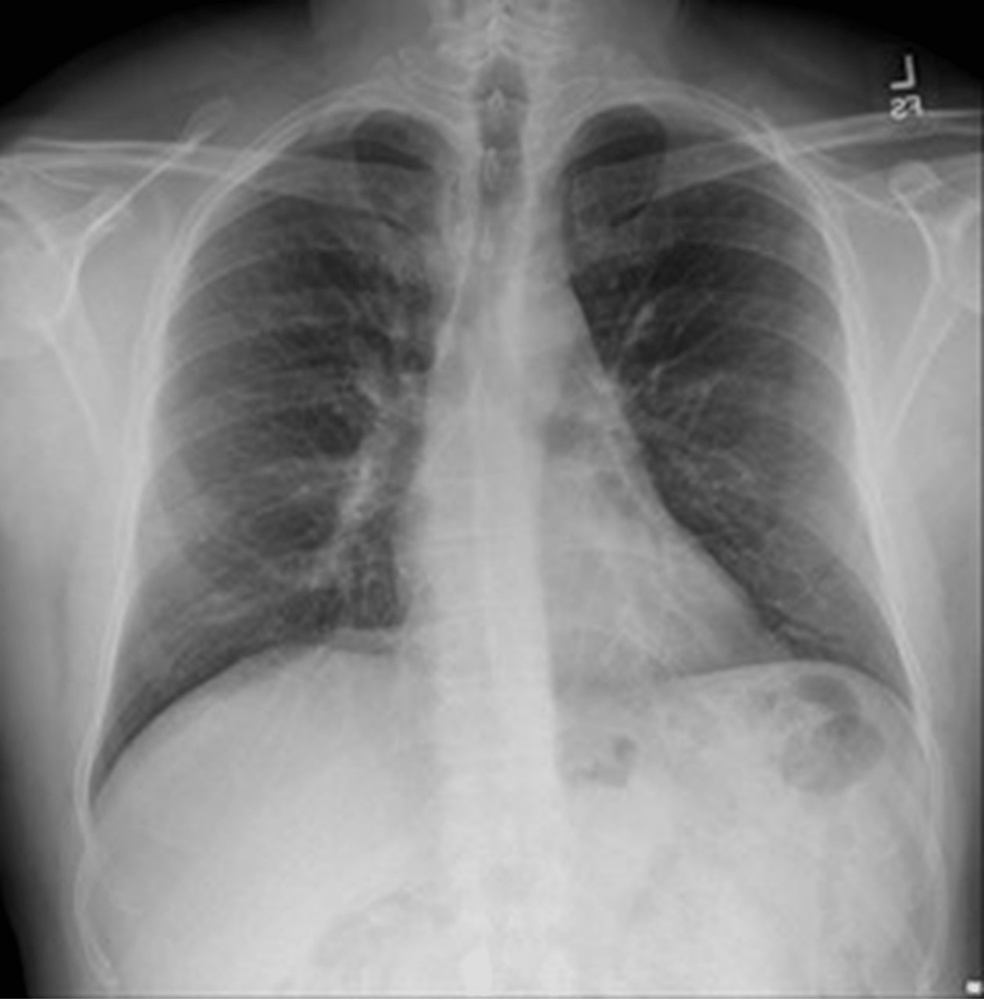
Chest X-ray of the patient during the first admission Note the absence of cardiopulmonary processes

Chest X-ray (CXR) (Figure 1) and computed tomography (CT) (data not shown) were negative for cardiopulmonary processes. The ECG was normal and the echocardiography was unremarkable. During an overnight stay, the patient remained afebrile, his leukocytosis, neutrophilia, and monocytosis were resolved, and troponin levels were normal on each of the three repeat tests ( as seen above in Table 1). Chest pain improved, blood pressure fluctuated around 125-126/75-80 mmHg, and mild tachycardia persisted (110-121 beats/minute). Based on available data, pulmonary embolism, pneumothorax, pneumonia, MI, pericardial disease, and aortic dissection were ruled out. The patient was discharged the following day with a diagnosis of bacterial bronchitis and was prescribed a five-day course of doxycycline 100 mg two times a day (BID) and dextromethorphan-guaifenesin every 12 hours (Q12H).

Case two 

**Figure 2:**
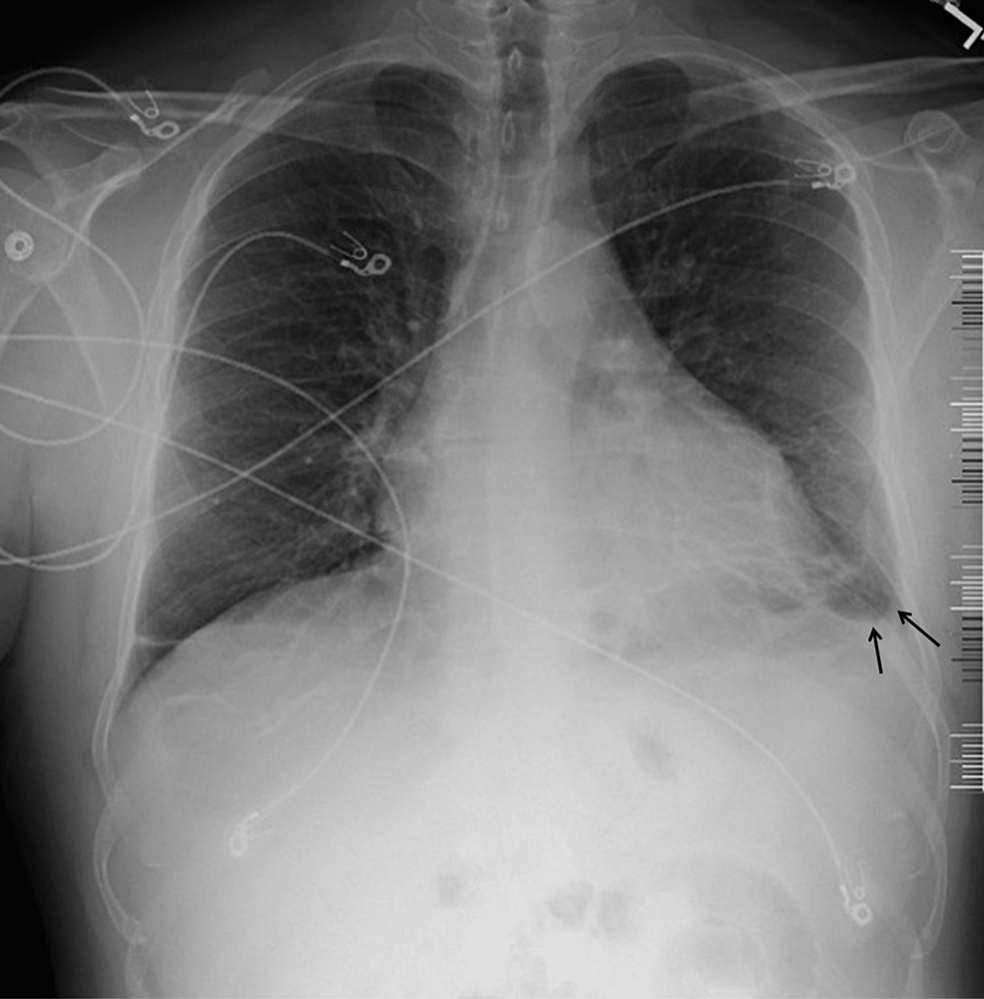
Chest X-ray of the patient during the second admission Lower left lobe infiltrates and minor pleural effusion (arrows) are seen. An enlarged cardiac silhouette is indicated by a cardiothoracic ratio of 0.6.

Although the patient’s chest pain had not returned to the severity that prompted the first admission, he continued to feel exhaustion most of the time and a consistent pattern emerged; he developed fevers [≥100.4˚F (38˚C)], chills, and extreme lethargy several minutes following physical exertion (e.g., lifting a heavy object or running upstairs). Less than three weeks after being discharged from his first hospitalization, he was diagnosed with influenza type B infection and received a five-day course of oseltamivir 75 BID. Despite treatment, the patient continued to have on-and-off episodes of fever, chills, and prostration; his chest pain, dyspnea, and tachycardia worsened; and his cough became productive of brownish or blood-tinged sputum. The patient returned to the emergency department almost one month after the first admission. On the second admission, the patient’s temperature was 98.9˚F (37.2˚C), heart rate was 132 beats/minute, respiratory rate was 16 breaths/minute, blood pressure was 157/90 mmHg, mean arterial pressure 112 mmHg, and oxygen saturation was 94% on room air. The CXR showed lower left lobe infiltrates and trace left pleural effusion. Heart size and mediastinal contours were noted to be normal in the radiology report, although upon careful examination and comparison to the CXR from the first admission, we found a cardiothoracic ratio of 0.6 (normal 0.42-0.50) [24]. Pulmonary vascularity was also noted as normal (Figure 2). Laboratory workup showed leukocytosis (16.6 109/L), neutrophilia (13.6 109/L, 82%), monocytosis (1.3 109/L), thrombocytosis (4.81 109/L), and elevated liver enzymes (alanine aminotransferase (ALT), aspirate aminotransferase (AST), and alkaline phosphatase of 159, 54, and 223 IU/mL, respectively) (Table 2). The patient was presumptively diagnosed with pneumonia and sepsis and was started on intravenous piperacillin/tazobactam 3.375 g every six hours (Q6H) and vancomycin (dose not available). Intravenous levofloxacin 750 mg QD was added the following day. 

**Table 2:** Laboratory investigation results on the first day of admission MCV: Mean corpuscular volume, MCH: Mean corpuscular hemoglobin, MCHC: Mean corpuscular hemoglobin concentration, ALT: Alanine aminotransferase, AST: Aspirate aminotransferase

Parameter	Results	Reference range
White blood cells (× 10^9^/L)	16.6	4.5-11.0
Red blood cells (× 10^12^/L)	4.85	4.3-5.9
Hemoglobin (g/dL)	14	13.5-17.5
Hematocrit (%)	42	41-53
MCV (fl)	86	80-100
MCH (pg)	29	25.4-34.6
MCHC (g/dL)	34	31%-36
Platelets (× 10^9^/L)	481	150-450
Neutrophils (× 10^9^/L)	13.6	2.5-8.0
Lymphocytes (× 10^9^/L)	1.1	1.0-4.0
Monocytes (× 10^9^/L)	1.3	0.1-0.7
Eosinophils (× 10^9^/L)	0.3	0.05-0.50
Basophils (× 10^9^/L)	0.0	0.025-0.100
Neutrophils (%)	82	55-70
Lymphocytes (%)	7	20-40
Monocytes (%)	8	2-8
Eosinophils (%)	2	1-4
Basophils (%)	0	0.5-1
ALT (IU/L)	159	9-46
AST (IU/L)	54	10-40
Alkaline phosphatase (IU/L)	223	44-147

**Figure 3:**
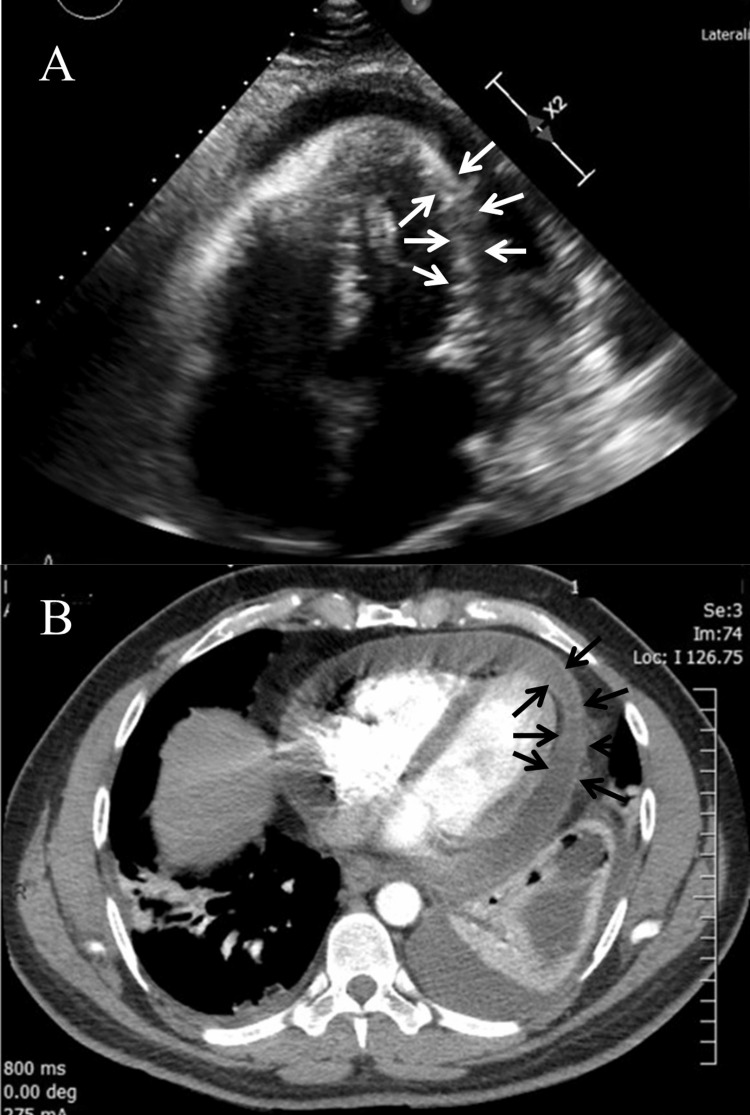
Echocardiography (A) and computed tomography (B) images taken on day one of the second admission. Pericardial effusion is evident in both echocardiography (white arrows) and computed tomography (black arrows).

On the day following admission (day one), the continuation of chest pain, shortness of breath, and tachycardia led to the ordering of an echocardiogram and a chest computed tomography angiography (CTA) with contrast (Omnipaque-350, 100mL), which revealed bibasilar consolidative infiltrations, atelectasis that was worse on the left side, bilateral pleural effusions, pericardial thickening, and a large pericardial effusion (Figure 3). The echocardiogram was negative for tamponade, and no evidence of pulmonary embolism was seen on CT. The patient was positive for *M. pneumoniae* immunoglobulin (Ig)G IgG at a level of 0.38, but negative for IgM. The patient was also seropositive for coxsackievirus and echovirus. Antibiotics were continued, and a cardiology consult was recommended. The patient declined thoracentesis and was prescribed furosemide 20 mg pending future scan results.

**Figure 4:**
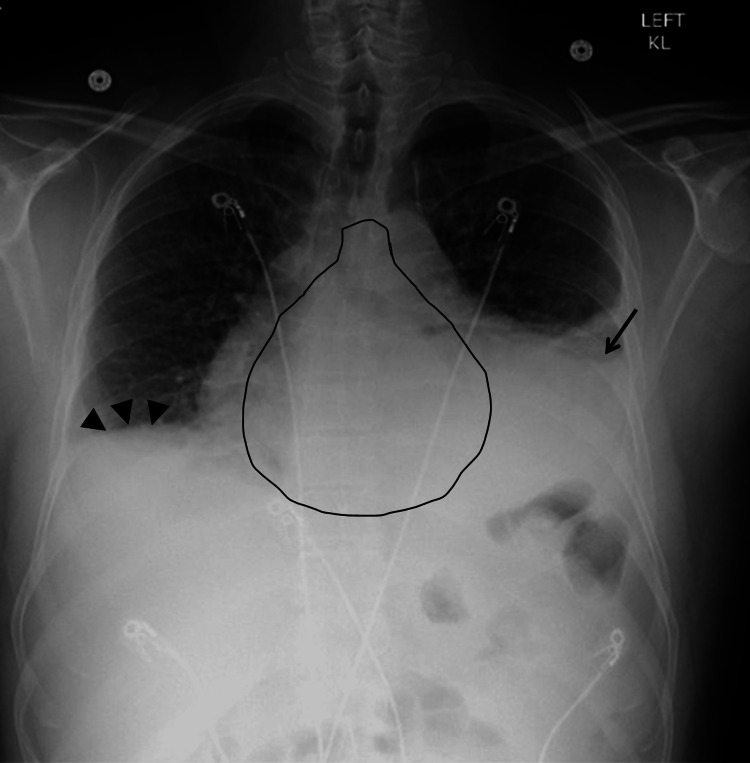
Chest X-ray of the patient on day three of the second admission Pericarditis with a water bottle sign (highlighted), left pleural effusion (arrow), left lower lobe pneumonia, and right lower lobe atelectasis (arrowheads) are seen.

On day three, the patient was found to have significantly decreased breath sounds in the left lower lung along with egophony changes. The CXR was ordered and showed a 'water bottle' presentation typical of effusive pericarditis. A worsening of the now moderate pleural effusion with left lower lobe infiltrates and right lower lobe atelectasis was noted (Figure 4).

Diagnosis

Based on the findings, the patient was diagnosed with pericarditis, left pleural effusion, left lower lobe pneumonia, right lower lobe atelectasis, and elevated liver enzymes.

Treatment

Due to acute viral pericarditis, the patient was prescribed high-dose ibuprofen 600 mg on prescription (t.i.d) and colchicine 0.6 mg for three months. For the duration of non-steroidal anti-inflammatory drug (NSAID) therapy, the patient was also prescribed proton pump inhibitor pantoprazole 3.375 gm intraventricular balloon pumping (IVBP) to control ibuprofen side effects. Ultrasound-guided thoracocentesis was performed on the left side of the chest to treat the patient’s atelectasis and pleural effusion. The procedure led to the draining of approximately 500 mL and immediate improvement in patient comfort and breathing. The CXR three days following thoracocentesis showed significantly decreased pleural effusion with only a small residue. The CXRs were continued daily until discharge, at which time the heart size had returned to normal and pleural effusion had resolved.

## Discussion

The main reason leading to the missed diagnosis of pericarditis on the first admission was the lack of CMR data. For the same reason, it remains unknown whether the inflammation had spread to the myocardium. Indeed, an enlarged cardiac silhouette was overlooked by the radiologist. However, it is often difficult to distinguish between possible diagnoses from a plain CXR, and if the elevated cardiothoracic ratio was noticed, a CMR would have still been needed to reach a definitive diagnosis. Early diagnosis of pericarditis and myopericarditis is critical. Delayed diagnosis may prolong the course of the pericardial disease and increase the risk of recurrence [11], and myocarditis poses more serious risks such as dilated cardiomyopathy and cardiac arrest [25]. Although troponin levels were within normal reference values during the several hours following the first presentation at the emergency department, normal troponin is not sufficient to rule out inflammation of the myocardium [26]. Therefore, after discharge from the first admission, the dyspnea and extreme fatigue experienced by the patient following extraneous physical activity could have also been indicative of myocardial involvement [25]. Not performing CMR on patients suspected of pericarditis or myopericarditis makes the diagnosis reliant on typical clinical presentation discoverable during the physical exam, ECG abnormalities, echocardiography, and CCT findings. As mentioned earlier, in the study by Mikolich et al., only 54.5% of patients with CMR-confirmed pericarditis showed diagnostic findings on ECG and 50% had a pericardial rub detectable on auscultation [12]. The same study also showed that out of the 29 patients who had CMR-detectable effusion, only five were detected by echocardiography [12]. This highlights the importance of CMR screening in patients suspected of having pericardial disease who could not be diagnosed by other more readily available technologies or have their symptoms explained by an alternative diagnosis. 

Laboratory-confirmed influenza type B infection acquired between the two admissions caused pericarditis exacerbation, eventually leading to the formation of pericardial effusion. However, the etiology of the initial pericardial disease remains unknown. Laboratory tests suggest multiple probable causes and further testing and had it been done during the acute illness and convalescence it could have helped better define the etiology. Based on serological evidence, the patient may have had a current or recent *M. pneumoniae* infection, which is a common cause of upper and lower respiratory infections, as well as cardiovascular infections, including pericarditis and myocarditis [18]. Although *M. pneumoniae* infections do not exhibit marked seasonality, outbreaks tend to occur in the summer and early fall [18], which is compatible with this case since the patient’s first presentation was at the beginning of October. Our patient tested positive for anti-*M. pneumoniae* IgG antibodies and negative for IgM antibodies. Typically, IgM antibodies are the first immunoglobulins elicited by exposure to antigens, whether due to vaccination or natural infection, via T-cell-independent responses [27]. Accordingly, an IgM+, IgG− test result is considered typical of acute infections, while an IgM−, IgG+ result is considered typical of previous or chronic infection. However, adults (above 25 years of age) tend to not elicit a detectable IgM response against *M. pneumoniae* [18], which makes the latter a possible etiologic agent in our case. On the other hand,* M. pneumoniae* is a common pathogen and IgG seropositivity is widespread among adult populations [18]. Therefore, available pertinent data cannot be used to prove or rule out *M. pneumoniae* infection as a definitive etiology. Optimally, an accurate determination of *M. pneumoniae* status is determined based on a rising titer (demonstrated by testing IgM and IgG titers in acute and convalescent specimens) along with polymerase chain reaction (PCR) detection of *M. pneumoniae* DNA [18]. Other probable etiologies include echovirus and coxsackievirus for which the patient was seropositive.

The patient believed his chronic allergies may have predisposed him to frequent upper respiratory infections in the few years leading to his hospitalization with pericarditis. Inflammation of the respiratory epithelia impedes mucociliary function and increases the risk of infection [28]. There have been reports in the literature linking pericarditis to antecedent upper respiratory tract infections [29-31]. A frequent history of allergies has also been reported [29]. The patient also reported physical exertion associated with interstate relocation four weeks before the first presentation. A study conducted in the 1950s concluded that a history of unusual physical or emotional exertion was “not infrequent” in a group of pericarditis patients who had no known diagnoses in the two weeks preceding the onset of pericarditis symptoms [29]. Since we could not find support for physical exertion being a risk factor for pericarditis in recent literature, we do not view physical exertion as a likely risk factor for pericarditis. However, we could not rule out the possibility due to insufficient evidence in the literature.

## Conclusions

We conclude that the patient had pericarditis on the first admission because other compatible causes of chest pain were ruled out, symptoms were compatible with non-effusive pericarditis and could not be ruled out since CMR was not performed, and the patient tested positive during his second admission for multiple known etiologic agents of pericarditis. We highlight the importance of CMR in screening patients presenting with chest pain of unknown origin, that is compatible with pericarditis, to facilitate early detection and intervention. Despite adequate data to definitively determine the origin of this patient’s pericarditis is lacking, we highlight some of the possible risk factors that have been supported by the literature and discuss the proper use of laboratory tests that could have assisted in defining the etiology of this case.
